# Associated factors of Nigerian Nurses' Emotion regulation, perceived stress, and coping mechanism during COVID-19 Pandamic: a cross-sectional study

**DOI:** 10.4314/ahs.v25i1.9

**Published:** 2025-03

**Authors:** Blessing Agbonselohbor, Aziz Aslanoğlu, Nurcan Bilgiç, Anas Alsharawneh, Rami A Elshatarat, Zyad T Saleh, Wesam T Almagharbeh, Hekmat Yousef Al-Akash, Dena E Sobeh, Mudathir M Eltayeb

**Affiliations:** 1 Department of Nursing, School of Health Sciences, Cyprus International University, Lefkosa, Turkish Republic of Northern Cyprus (TRNC); 2 Department of Nursing, Vision College, Riyadh, Saudi Arabia; 3 Department of Nursing, School of Health Sciences, Cyprus World Peace University, Lefkosa, Turkish Republic of Northern Cyprus (TRNC); 4 Department of Adult Health Nursing, Faculty of Nursing, The Hashemite University, Zarqa, Jordan; 5 Department of Medical and Surgical Nursing, College of Nursing, Taibah University, Madinah, Saudi Arabia; 6 Department of Clinical Nursing, School of Nursing, The University of Jordan, Amman, Jordan; 7 Medical Surgical Nursing Department, Faculty of Nursing, University of Tabuk, Tabuk, Saudi Arabia; 8 Faculty of Nursing, Applied Science Private University, Amman, Jordan; 9 Department of Medical Surgical Nursing, College of Nursing, Prince Sattam bin Abdulaziz University, AlKharj, Saudi Arabia

**Keywords:** COVID-19 pandamic, stress, emotional regulation, coping mechanisms, socio-demographic factors, work-related factors, Nigeria

## Abstract

**Background:**

Nursing, marked by its demanding nature, often exposes professionals to elevated stress levels and emotional hurdles. Recognizing the determinants shaping nurses' stress, emotional regulation, and coping mechanisms is imperative for fostering their welfare and maintaining superior patient care standards.

**Objective:**

This study aimed to explore the correlation between socio-demographic and work-related factors and nurses' perceived stress, emotional regulation, and coping mechanisms.

**Methods:**

A cross-sectional study encompassing 200 nurses in Nigeria was conducted. Participants completed questionnaires evaluating socio-demographic details, the Perceived Stress Scale (PSS), Emotion Regulation Questionnaire (ERQ), and Coping Mechanism Scale. Statistical analyses, comprising t-tests and ANOVA, were employed to scrutinize variable associations.

**Results:**

Participants exhibited a mean score of 27.1 (±3.7) (out of 40) on the PSS, indicating heightened stress levels. Notably, cognitive reappraisal scored 32.2 (±5.2) (out of 42) on the ERQ, while expressive suppression scored 23.4 (±3.8) (out of 28), with a total ERQ score averaging 55.5 (±9.8) (out of 70), indicating moderate emotion regulation. Furthermore, participants scored 78.1 (±10.7) (out of 112) on the Coping Mechanism Scale, suggesting frequent utilization of coping strategies. While gender, age, religion, and employment status showed no significant correlations with stress levels, factors such as educational attainment, number of children, years of experience, department worked in, job position, and work environment satisfaction displayed noteworthy relationships. Various socio-demographic and work-related factors, including the number of children, years of experience, department worked in, and job position, demonstrated significant relationships with nurses' emotional experiences and coping strategies.

**Conclusion:**

The study underscores the intricate interplay between socio-demographic and work-related factors in shaping nurses' stress, emotional regulation, and coping mechanisms. Interventions tailored to address these factors and cultivate supportive work environments are vital for safeguarding nurses' well-being and upholding exceptional patient care standards.

## Introduction

The emergence of the COVID-19 pandemic has posed an unprecedented global health crisis, severely straining healthcare systems worldwide^1^. Nurses, forming the backbone of the medical workforce, have been thrust onto the frontlines of this battle, providing critical care to patients often in close proximity, placing themselves at increased risk of infection^1^, ^2^. This highly contagious and potentially lethal virus has wreaked havoc on social, financial, and healthcare systems, presenting a significant physical and psychological threat to healthcare workers' well-being^1^-^3^.

This study focuses on emotion regulation, perceived stress, and coping mechanisms as primary variables, as they are vital for comprehending the psychological well-being of nurses, particularly during crises like the COVID-19 pandemic^3^-^7^. Emotion regulation is crucial for nurses, as it directly affects their ability to manage stress and maintain professionalism under pressure^8^-^10^. Perceived stress reflects how nurses experience and respond to the demands of their jobs, impacting both their mental health and job performance. Coping mechanisms are essential for mitigating the adverse effects of stress, enabling nurses to continue providing quality care under challenging conditions. Together, these variables form a comprehensive framework for assessing the mental health and resilience of nurses, making them essential to this study^1^,^4^,^11^. İn detail, emotion, as elucidated by Charles Darwin and corroborated by contemporary research, serves as a vital communication tool and survival trait, particularly in high-pressure environments such as healthcare settings 8. Professional responsibilities inherent in nursing practice are often accompanied by emotional constraints, which can cascade into various stressors impacting nurses' well-being 10. Perceived stress, defined by Lazarus and Folkman (1984), encompasses individuals' reactions to environmental threats and challenges, a phenomenon accentuated in the healthcare context where nurses confront physical and social consequences of diseases daily 4, 12-14. Coping mechanisms represent essential strategies employed by nurses to navigate stressors, enabling them to approach challenges optimistically and mitigate stress effectively^1^,^4^,^11^,^15^.

Despite existing research on the psychological impact of infectious disease outbreaks on healthcare professionals, including nurses, a significant gap remains in understanding the specific experiences of Nigerian nurses during the COVID-19 pandemic 16-18. The unique socio-cultural, economic, and healthcare landscape of Nigeria likely influences how nurses in the country manage their emotions, perceive stress, and cope with the challenges introduced by the pandemic. Understanding these factors in the Nigerian context is crucial for developing targeted interventions to support nurses' well-being 17, 18. Furthermore, it is essential to explore the specific challenges Nigerian nurses faced during the COVID-19 pandemic. These challenges include frequent exposure to death and severe illness, leading to increased emotional and psychological stress, extended working hours under strenuous conditions, and persistent shortages of personal protective equipment (PPE)^6^,^17^,^18^. These factors have significantly affected Nigerian nurses' ability to manage stress, regulate emotions, and cope with their demanding roles, highlighting the importance of addressing these issues in the study. Moreover, the stress and shortage of nurses can lead to significant challenges, including the need for nurses to take on tasks outside their typical roles^3^, ^12^, ^19^, ^20^. This issue is explored in studies by Al-Akash et al.^19^ and Aldarawsheh et al.^20^, which reveal that nurses often perform non-nursing tasks due to workforce shortages, contributing to increased stress and job dissatisfaction.

These studies emphasize the importance of addressing nurse shortages and streamlining job responsibilities to prevent burnout and ensure that nurses can focus on their primary care duties. By alleviating the burden of non-nursing tasks, healthcare systems can improve job satisfaction and retention rates, ultimately leading to better patient care outcomes^16^, ^19^-^21^.

Furthermore, international studies have highlighted the profound impact of the COVID-19 pandemic on healthcare workers, particularly in terms of job satisfaction, stress, and coping mechanisms^6^, ^15^, ^22^, ^23^. For instance, Yasin et al. 23 explored job satisfaction among expatriate nurses during the pandemic, emphasizing the importance of enhancing nursing programs to better prepare nurses for such crises Similarly, Kehyayan et al. 24 investigated the fear and stress related to COVID-19 among undergraduate nursing students, underscoring the need for culturally tailored educational interventions. Elneblawi et al. 6 focused on nurses' perceptions of exposure to COVID-19 risks and the psychosocial impact of the outbreak, further reinforcing the necessity of improving nursing curricula and satisfaction levels to better equip nurses for the challenges posed by pandemics. These studies collectively suggest that a robust and well-structured nursing education program, coupled with strategies to enhance job satisfaction, is crucial in fostering resilience and effective coping mechanisms among nurses during global health crises.

This study addresses a critical research gap by focusing on the psychological well-being of Nigerian nurses during the COVID-19 pandemic. While previous research has examined various aspects of healthcare workers' mental health globally, there is a distinct lack of studies specifically exploring the unique challenges faced by Nigerian nurses^17^,^18^. The heightened stress levels due to increased exposure to death and illness, long working hours, and inadequate PPE during the pandemic exacerbate these challenges. Therefore, this study investigates the emotional responses, perceived stress levels, and coping strategies employed by Nigerian nurses during the COVID-19 pandemic. By addressing this knowledge gap, the findings can inform targeted interventions and policies to strengthen nurses' mental health and resilience^25^, ^26^, ultimately enhancing the capacity of the Nigerian healthcare system to respond effectively to ongoing challenges posed by the pandemic.

### Study Purpose and Objectives

This study aims to explore the complex relationships between emotional responses, perceived stress, and coping strategies among nurses. The specific objectives of this research are to: 1) assess the levels of perceived stress, emotion regulation, and coping mechanisms among the participants; 2) examine the relationship between nurses' socio-demographic and work-related characteristics and their perceived stress levels; 3) investigate the connection between nurses' socio-demographic and work-related characteristics and their emotional regulation; and 4) explore the relationship between nurses' socio-demographic and work-related characteristics and their coping strategies. Understanding the interplay between these factors is essential for gaining a comprehensive understanding of nurses' mental health. By using a cross-sectional design, this study provides a snapshot of these dynamics at a specific point in time, laying the groundwork for future longitudinal studies to track changes and developments over time.

## Methodology

### Study Design

This study utilized a quantitative cross-sectional design to investigate the associated factors of emotion, perceived stress, and coping mechanisms among Nigerian nurses during the COVID-19 pandemic. By employing a cross-sectional design, this study aims to identify and understand the factors associated with emotion, perceived stress, and coping mechanisms among Nigerian nurses during the COVID-19 pandemic. This approach will provide valuable insights into the psychological well-being of nurses and inform interventions aimed at supporting their mental health during challenging times.

### Setting and Sample

The study was conducted at two prominent general hospitals located in Lagos and Abuja, Nigeria, chosen for their extensive range of medical and nursing services designed to meet the diagnostic and treatment needs during the COVID-19 pandemic. These hospitals were strategically selected due to their advanced medical facilities and the significant role they played in addressing the complex challenges posed by the pandemic. They served as key centers for frontline healthcare, providing an ideal environment to study the experiences of nurses directly involved in COVID-19 patient care.

A convenience sampling method was used to recruit 200 nurses, with an equal distribution of 100 nurses from each hospital. This approach ensured the inclusion of a diverse group of participants who were actively engaged in patient care during the pandemic. The sample consisted of registered nurses who met specific inclusion criteria: direct patient contact and a minimum of one year of work experience. Nurses not directly involved in patient care or with less than one year of experience were excluded, as were those who had been diagnosed with COVID-19 or psychological disorders during the study period.

The participants in the study varied in age, gender, years of experience, and specialty. The age of the nurses ranged from 23 to 55 years. The sample included both male and female nurses, though the majority were female, reflecting the general gender distribution in the nursing profession in Nigeria. The participants had diverse years of experience, ranging from 1 to over 20 years, providing a broad perspective on how different levels of experience influenced their emotional regulation, perceived stress, and coping mechanisms. Additionally, the nurses represented various specialties, including emergency care, intensive care, general medicine, and pediatrics. This diversity in the sample enhances the generalizability of the findings, offering insights into how different demographic and work-related factors influence nurses' mental health during a global health crisis.

The effective sample size for the study was determined utilizing G*Power software, configuring the analysis for either a t-test or ANOVA test (based on the nature of the variables). With a moderate effect size of 0.30, a significance level (α) of 0.05, and a power (1-β) of 80%, the calculated effective sample size was found to be minimally 184. Despite this, the authors distributed 234 questionnaires to potential participants. Ultimately, 200 completed questionnaires were received and included in the final data analysis, ensuring the acquisition of a robust dataset conducive to thorough statistical analysis and interpretation.

### Instruments

In this study, data collection was facilitated through a comprehensive set of instruments tailored to gather essential information on various aspects of the participants' psychological well-being amidst the COVID-19 pandemic.

### Socio-demographic Information

The first instrument employed was a socio-demographic information form comprising 19 questions meticulously crafted to capture a range of socio-demographic details. These questions delved into key areas such as age, gender, educational background, marital status, monthly income, employment status, and years of experience in the nursing profession.

### Perceived Stress Scale (PSS)

The PSS stands as a widely recognized and reliable psychological instrument employed to gauge individuals' perceptions of stress 27, 28. Originating from the work of Cohen, Kamarck, and Mermelstein in 1983, this scale encompasses 10 questions aimed for assessing the frequency of stress-inducing situations encountered in individuals' lives^27^. Participants rated their responses on a 5-point Likert scale, ranging from “Never” to “Very Often.” Scores on the PSS ranged from 0 to 40, with higher scores indicating higher levels of perceived stress among participants 27.

Individual scores on the PSS can range from 0 to 40, with higher scores reflecting higher perceived stress levels. Scores falling within the range of 0-13 are classified as low stress, while scores between 14 and 26 indicate moderate stress. Scores from 27 to 40 are indicative of high perceived stress levels^27^,^28^.

### Coping Mechanisms Scale

To gain a deeper understanding of participants' coping mechanisms, we utilized the Coping Mechanisms Scale, which comprises 28 questions designed to assess the frequency and effectiveness of various coping strategies employed by participants 29. Developed by Carver in 1997, this scale allows individuals to indicate the extent to which they engage in specific coping behaviors using a 4-point Likert scale. Responses range from “I haven't been doing this at all” to “I've been doing this a lot.” Scores on the scale range from 1 to 4, with higher scores indicating a more frequent use of coping strategies 29.

### Emotion Regulation Questionnaire (ERQ)

The ERQ is a psychological instrument designed to assess individuals' habitual use of specific emotion regulation strategies, particularly cognitive reappraisal and expressive suppression 30, 31. Developed by Gross and John in 2003, the ERQ is rooted in Gross's process model of emotion regulation, which categorizes strategies based on their timing and consequences in the emotion generation process^30^, ^31^.

The ERQ consists of 10 items, each representing a statement related to how individuals regulate their emotions. These items are designed to measure the frequency with which respondents engage in cognitive reappraisal and expressive suppression. Cognitive reappraisal involves changing one's cognitive interpretation of an emotional event to modify its emotional impact, while expressive suppression involves inhibiting the outward manifestation of one's emotions^30^, ^31^.

Each item on the ERQ is rated on a 7-point Likert scale, ranging from “Strongly Disagree” to “Strongly Agree.” Respondents indicate the extent to which they agree with each statement based on their typical behavior or tendency. Higher scores on the ERQ indicate a greater propensity to employ the respective emotion regulation strategy^30^,^31^.

The ERQ provides valuable insights into individuals' emotion regulation tendencies and preferences. By understanding how individuals regulate their emotions, researchers and practitioners can gain a deeper understanding of psychological functioning, coping mechanisms, and emotional well-being. Additionally, the ERQ has been widely used in various research domains, including clinical psychology, social psychology, and affective neuroscience, to investigate emotion regulation processes across different populations and contexts. Overall, the ERQ is a reliable and valid tool for assessing emotion regulation strategies and has contributed significantly to our understanding of how individuals manage their emotions in everyday life^30^, ^31^.

Collectively, these instruments facilitated a comprehensive assessment of participants' socio-demographic characteristics, perceived stress levels, coping mechanisms, and emotion regulation strategies. By delving into these key domains, the study aimed to deepen our understanding of the psychological well-being of nurses during the COVID-19 pandemic, thus contributing to the broader discourse on healthcare professionals' mental health and resilience in times of crisis.

### Data Collection Procedure

The data collection procedure for this study was designed with careful attention to detail to ensure rigorous recruitment, informed consent, and data confidentiality. The process involved several structured steps to uphold the research's integrity and ethical standards.

The data collection began with the researchers obtaining a comprehensive list of eligible nurses, along with their duty schedules, from the head nurses at the selected hospitals in Lagos and Abuja. This list was crucial in identifying nurses who met the inclusion criteria of having direct patient contact and at least one year of work experience. With this information, a designated co-author responsible for data collection reached out personally to the eligible nurses. During these initial contacts, the co-author conducted interviews to explain the study's objectives, procedures, potential risks, and benefits, ensuring that the nurses fully understood what participation entailed. Any questions or concerns raised by the nurses were addressed promptly.

For those interested in participating, detailed information sheets were provided, outlining the study's purpose, procedures, confidentiality measures, and contact information for the research team. This documentation enabled nurses to make an informed decision about their involvement. Following the provision of this information, consent forms were distributed to those who agreed to participate. These forms detailed the voluntary nature of participation, guaranteed the confidentiality of responses, and reaffirmed participants' rights to withdraw from the study at any time without consequence. Signed consent forms were collected and securely stored to ensure privacy and confidentiality.

Once consent was obtained, participants completed the study instruments, including a socio-demographic information form, the PSS, the Coping Mechanisms Scale, and the ERQ. The co-author provided clear instructions for completing these instruments and was available to answer any questions during the process.

Data collection continued until the target sample size of 200 participants was reached. Throughout the data collection process, strict measures were implemented to maintain ethical standards and protect participants' rights, ensuring the reliability and confidentiality of the data gathered.

## Ethical Considerations

Upholding ethical standards was paramount throughout the study, with rigorous measures enacted to safeguard participants' rights and well-being. This involved obtaining formal approval from the Institutional Review Board (IRB) at Cyprus International University (Ethical Application Ref: Nsg-21-018) and relevant IRBs of selected hospital settings. Additionally, ethical letters were secured from nursing administrators, reflecting institutional support, and facilitating letters ensured smooth access to hospital premises.

Participants were provided with detailed information about the study, including its objectives, procedures, and potential risks and benefits, allowing for informed decisions. Informed consent was obtained from each participant, emphasizing voluntariness and confidentiality. Stringent measures, such as anonymizing participant identities and securely storing data, were implemented to protect confidentiality. By adhering to these ethical considerations, the study aimed to uphold principles of beneficence, respect for autonomy, and justice, ensuring the integrity and credibility of its findings while prioritizing participant well-being.

### Data Analysis

Statistical analysis was conducted using SPSS 20.0 (Statistical Package for the Social Sciences) to explore and interpret the collected data. The analysis involved a comprehensive approach to examining various aspects of the data, employing both descriptive and inferential statistical techniques.

Descriptive statistics were utilized to summarize and present the data in a clear and concise manner. This included calculating measures such as numbers, percentages, means, standard deviations, and medians to provide insights into the characteristics and distribution of variables within the sample. Descriptive statistics facilitated the understanding of key trends and patterns observed in the data, aiding in the interpretation of results.

In addition to descriptive analysis, inferential statistical tests were employed to assess relationships and differences between variables. Specifically, the t-test or ANOVA tests were utilized to compare different groups within the sample, examining potential differences in perceived stress levels, coping mechanisms, and emotion regulation strategies among nurses based on various socio-demographic factors. These statistical tests allowed for the identification of significant associations and differences, contributing to a deeper understanding of the factors influencing nurses' experiences during the COVID-19 pandemic.

## Results

### Socio-demographic and Work-related Data

Table 1 presents socio-demographic and work-related data of 200 participants involved in the study. The sample consisted of predominantly female participants, comprising 61.0% of the total, with males representing 39.0%. In terms of age distribution, 38.5% of participants were under the age of 30, while 61.5% were 30 years old or above. Regarding religious affiliation, the majority of participants identified as Christians, accounting for 69.5% of the sample, while 30.5% identified as Muslims. Regarding educational attainment, 67.5% of participants held either an Associate or Bachelor's degree, while 32.5% held a Postgraduate (Master or PhD) degree. In terms of employment status, the majority of participants were employed full-time, comprising 72.0% of the sample, while 28.0% were employed part-time. Concerning the number of children, 44.5% of participants had between 0 to 2 children, whereas 55.5% had more than 2 children. When examining years of working as a nurse in the hospital, 58.5% of participants had been working for 1-5 years, while 41.5% had been working for more than 5 years. In terms of departmental distribution, a majority of participants worked in other departments (77.0%), followed by those working in the Intensive Care Unit (10.5%) and the Emergency Department (12.5%). Regarding working hours per day, 62.0% of participants reported working more than 8 hours per day, with 18.0% working morning shifts, 62.0% working afternoon shifts, and 20.0% working night shifts. Lastly, in terms of job position, the majority of participants were licensed practical nurses. Regarding satisfaction with teammates in the working environment, 65.0% reported being always satisfied, 3.5% reported never being satisfied, and 31.5% reported being sometimes satisfied. Similarly, regarding the status of being appreciated by superiors, 73.0% reported always being appreciated, 1.5% reported never being appreciated, and 25.5% reported being sometimes appreciated. Additionally, regarding estrangement to their profession, 68.0% reported no estrangement, 14.5% reported partial estrangement, and 17.5% reported complete estrangement.

**Table 1:** Socio-demographics and work-related data (N= 200)

Variables	n (%)
**Gender**	
Female	122 (61.0%)
Male	78 (39.0%)
**Age**	
< 30	77 (38.5%)
≥ 30	123 (61.5%)
**Religion**	
Christianity	139 (69.5%)
Islam	61 (30.5%)
**Level of Education**	
Associate or Bachelor Degree	135 (67.5%)
Postgraduate (Master or PhD) Degree	65 (32.5%)
**Employment Status**	
Employed Full-Time	144 (72.0%)
Employed Part-Time	56 (28.0%)
**Number of Children**	
0 - 2	89 (44.5%)
> 2	111 (55.5%)
**Years of working as a Nurse in the hospital**	
1-5 years	117 (58.5%)
> 5 years	83 (41.5%)
**Department working as a Nurse in the hospital**	
Emergency department	25 (12.5%)
Intensive care unit	21 (10.5%)
Other departments	154 (77.0%)
**Working Hours per day**	
≤8 hours	141 (70.5%)
> 8 hours	59 (29.5%)
**Current job rotation status**	
Morning shift	36 (18.0%)
Afternoon shift	124 (62.0%)
Night shift	40 (20.0%)
**Job Position**	
Licensed practical nurse	136 (68.0%)
Nurse practitioner	64 (32.0%)
**How satisfied are you with your teammates in your working environment?**	
Always	130 (65.0%)
Never	7 (3.5%)
Sometimes	63 (31.5%)
**Status of being appreciated by superiors**	
Always	146 (73.0%)
Never	3 (1.5%)
Sometimes	51 (25.5%)
**Estrangement to their profession**	
No	136 (68.0%)
Partially	29 (14.5%)
Yes	35 (17.5%)

### Participants' Perceptions of Stress, Emotion Regulation, and Coping Mechanisms

Table 2 presents an overview of participants' perspectives regarding stress, emotion regulation, and coping mechanisms. Regarding the total score of PSS, participants indicated a mean score of 27.1 (±3.7). Scores ranging from 27-40 were deemed to signify high perceived stress levels, suggesting that, on average, participants experienced high stress levels. The ERQ assessed two main sub-scales: Cognitive reappraisal and Expressive suppression. Participants reported a mean score of 32.2 (±5.2) for cognitive reappraisal, indicating a tendency to utilize cognitive strategies for emotion regulation. Additionally, a mean score of 23.4 (±3.8) was reported for expressive suppression, suggesting a higher usage of this strategy among participants. The total ERQ score, combining both sub-scales, averaged at 55.5 (±9.8), signifying an overall inclination towards employing emotion regulation strategies. In terms of coping mechanisms, participants scored an average of 78.1 (±10.7) on the Coping Mechanism Scale. This score indicates a frequent utilization of coping strategies to address stressors and challenges.

**Table 2:** Participants' perceptions of stress, emotion regulation, and coping mechanisms

Variables	Mean (SD)	Interpretation
**Total score of Perceived Stress Scale (PSS) (out of 40)**	27.1 (±3.7)	Scores ranging from 27-40 would be considered high perceived stress
**Emotion Regulation Questionnaire (ERQ)**		
Cognitive reappraisal sub-scale (6 items) (out 42)	32.2 (±5.2)	Higher scores indicating higher usage of emotion regulation strategy
Expressive suppression sub-scale (4 items) (out of 28)	23.4 (±3.8)	Higher scores indicating higher usage of emotion regulation strategy
Total score of ERQ (10 items) (out of 70)	55.5 (±9.8)	Higher scores indicating higher usage of emotion regulation strategy
**Coping Mechanism Scale (28 items) (out of 112)**	78.1 (±10.7)	Higher scores indicating more frequent utilization of coping mechanism strategies

Overall, the findings suggest that participants generally employed emotion regulation and coping mechanisms to manage stress. Higher scores on the cognitive reappraisal sub-scale of the ERQ imply a preference for cognitive strategies in regulating emotions. Additionally, the moderate score on the PSS indicates a high level of perceived stress, while the high score on the coping mechanism scale reflects active engagement in coping behaviors. These insights into participants' emotional experiences and coping strategies could inform interventions aimed at enhancing psychological well-being and stress management.

### Associated factors of perceived stress

Table 3 provides insights into the relationship between various socio-demographic and work-related factors and perceived stress levels among nurses. Firstly, the analysis by gender indicates a slightly higher mean stress score for female nurses (27.8) compared to male nurses (26.1), though the difference was not statistically significant (p= 0.062). Similarly, age, religion, and employment status did not show significant associations with perceived stress levels. However, certain factors displayed noteworthy relationships with perceived stress. Nurses with an associate or bachelor degree reported significantly higher stress levels (mean=28.3) compared to those with a postgraduate degree (mean=24.5), with a p value <0.001. Moreover, nurses with more than two children had significantly higher stress levels (mean=27.5) compared to those with fewer children (p <0.001). Similarly, nurses with over 5 years of experience, those working in the Emergency department, and those on night shifts exhibited significantly higher stress levels (p <0.001). Additionally, job position and work environment satisfaction had significant associations with perceived stress (p <0.001). Licensed practical nurses reported significantly higher stress levels (mean=28.2) compared to nurse practitioners (mean=23.6), with a p value <0.001. Nurses who reported being always satisfied with their teammates or always appreciated by their superiors also exhibited significantly higher stress levels compared to their counterparts (p <0.001). However, some factors like feeling estranged from the profession did not show statistically significant associations with perceived stress levels (p= 0.1623). These findings underscore the complex interplay between various socio-demographic and work-related factors in influencing nurses' stress levels, highlighting potential areas for intervention and support to mitigate stress in the nursing workforce.

**Table 3:** Relationship between nurses' socio-demographics and work-related characteristics and perceived stress

Variables	Mean (SD)^	t-value or f-value	*P* value*
**Gender**			
Female	27.8 ± 6.0	1.87	0.062
Male	26.1 ± 6.7		
**Age**			
< 30	26.9 ± 5.6	1.23	0.294
≥ 30	26.3 ± 7.2		
**Religion**			
Christianity	27.2 ± 5.8	1.37	0.172
Islam	25.8 ± 7.9		
**Level of education**			
Associate or Bachelor degree	28.3 ± 4.8	4.08	**<0.001**
Postgraduate (Master or PhD) degree	24.5 ± 8.9		
**Employment status**			
Employed full-time	28.9 ± 11.4	1.66	0.218
Employed part-time	23.6 ± 10.9		
**Number of children**			
0 - 2	23.1 ± 5.3	19.97	**<0.001**
> 2	27.5 ± 6.8		
**Years of working as a nurse in the hospital**			
1-5 years	26.1 ± 5.1	4.31	**<0.001**
> 5 years	30.1 ± 4.7		
**Department working as a nurse in the hospital**			
Emergency department	30.1 ± 2.8	4.83	**0.006**
Intensive care unit	28.9 ± 5.3		
Other departments	23.3 ± 5.5		
**Working hours per day**			
≤8 hours	23.2±4.7	3.31	**0.001**
> 8 hours	26.6±5.9		
**Average number of patient are cared for per day**			
≤ 5 patients	21.5 ± 3.7	24.16	**<0.001**
> 5 patients	28.4 ± 4.2		
**Current job rotation status**			
Morning shift	25.9 ± 6.73	57.49	**0.048**
Afternoon shift	26.0 ± 7.12		
Night shift	29.8 ± 3.93		
**Job position**			
Licensed practical nurse	28.2 ± 5.5	16.22	**<0.001**
Nurse practitioner	23.6 ± 4.2		
**How satisfied are you with your teammates in your working environment?**			
Always	27.6 ± 6.8	4.32	**<0.001**
Never	21.9 ± 6.5		
Sometimes	25.5 ± 5.7		
**Status of being appreciated by superiors**			
Always	28.1 ± 6.3	15.68	**<0.001**
Never	15.0 ± 7.1		
Sometimes	23.6 ± 5.4		
**Do you feel estranged from your profession?**			
No	26.5 ± 6.4	1.83	0.1623
Partially	25.7 ± 5.6		
Yes	28.6 ± 7.3		

^ Higher scores indicating higher perceived stress

* Significant *p* value (< 0.05) is bold

### Associated Influencing Factors for Improving Participants' Emotional Regulation

Table 4 provides insights into the relationship between various socio-demographic and work-related factors and the utilization of emotional regulation strategies among nurses. Gender did not show a significant difference in emotional regulation scores, with female nurses scoring slightly lower than male nurses, but the variation was not statistically significant. Similarly, age and religion also did not exhibit significant associations with emotional regulation scores among nurses.

**Table 4:** Relationship between nurses' socio-demographics and work-related characteristics and emotional regulation

Variables	Mean (SD)^	t-value or f-value	*P* value*
**Gender**			
Female	54.9 ± 9.4	0.77	0.440
Male	56.0 ± 9.8		
**Age**			
< 30	55.9 ± 9.5	0.21	0.807
≥ 30	55.2 ± 9.9		
**Religion**			
Christianity	55.4 ± 9.7	0.23	0.818
Islam	55.8 ± 9.4		
**Level of education**			
Associate or Bachelor degree	55.7 ± 10.5	18.04	0.148
Postgraduate (Master or PhD) degree	56.9 ± 7.5		
**Employment status**			
Employed full-time	56.4 ± 11.4	1.81	0.338
Employed part-time	54.1 ± 10.9		
**Number of children**			
0 - 2	52.3 ± 14.1	8.42	**<0.001**
> 2	57.9 ± 4.5		
**Years of working as a nurse in the hospital**			
1-5 years	52.0 ± 13.6	4.26	**0.003**
> 5 years	58.8 ± 7.4		
**Department working as a nurse in the hospital**			
Emergency department	58.0 ± 9.5	1.47	0.159
Intensive care unit	55.6 ± 11.9		
Other departments	56.6 ± 11.4		
**Working hours per day**			
≤8 hours	54.3 ± 9.6	18.51	0.139
> 8 hours	55.8 ± 9.6		
**Average number of patient are cared for per day**			
≤ 5 patients	54.8 ± 8.6	27.43	**<0.001**
> 5 patients	50.2 ± 14.4		
**Current job rotation status**			
Morning shift	55.3 ± 9.6	0.14	0.865
Afternoon shift	56.3 ± 9.4		
Night shift	55.5 ± 9.8		
**Job position**			
Licensed practical nurse	55.4 ± 9.4	0.02	0.465
Nurse practitioner	55.7 ± 8.9		
**How satisfied are you with your teammates in your working environment?**			
Always	56.7 ± 7.3	33.42	**0.004**
Never	49.0 ± 16.7		
Sometimes	53.9 ± 12.0		
**Status of being appreciated by superiors**			
Always	56.4 ± 8.1	36.64	**0.003**
Never	63.0 ± 4.4		
Sometimes	52.7 ± 12.6		
**Do you feel estranged from your profession?**			
No	55.0 ± 8.1	14.28	0.242
Partially	55.3 ± 4.4		
Yes	58.1 ± 12.6		

^ Higher scores indicating higher usage of emotional regulation strategy

* Significant p value (< 0.05) is bold

However, certain factors displayed notable relationships with emotional regulation. While nurses' level of education did not show a significant difference in emotional regulation scores, factors such as the number of children, years of experience as a nurse, working hours per day, and average number of patients cared for per day exhibited significant associations. Nurses with more than two children demonstrated significantly higher emotional regulation scores compared to those with fewer children, while those with longer years of experience tended to have higher emotional regulation scores. Additionally, nurses who cared for fewer patients per day and those who reported being always satisfied with their teammates or always appreciated by their superiors showed significantly higher emotional regulation scores. These findings underscore the importance of factors such as workload, interpersonal relationships, and support systems in influencing nurses' emotional regulation strategies.

### Associated Influencing Factors for Improving Participants' Coping Mechanism

The findings from Table 5 provide insights into the relationship between nurses' socio-demographic and work-related characteristics and their utilization of coping mechanism strategies. Starting with gender, female nurses exhibited a slightly higher mean score for coping mechanisms (78.40 ± 9.80) compared to male nurses (78.12 ± 10.89), but this difference was not statistically significant (t-value = 0.237, p = 0.8126). Similarly, age, religion, and level of education did not show significant associations with coping mechanism strategies.

**Table 5:** Relationship between nurses' socio-demographics and work-related characteristics and coping mechanism strategy

Variables	Mean (SD)^	t-value or f-value	*P* value*
**Gender**			
Female	78.40 ± 9.80	0.23	0.8126
Male	78.12 ± 10.89		
**Age**			
< 30	78.12 ± 10.08	0.07	0.9262
≥ 30	77.84 ± 11.76		
**Religion**			
Christianity	77.84 ± 10.64	0.78	0.9720
Islam	79.24 ± 9.80		
**Level of education**			
Associate or Bachelor degree	78.96 ± 10.08	0.91	0.4361
Postgraduate (Master or PhD) degree	79.91 ± 8.96		
**Employment status**			
Employed full-time	80.64 ± 8.40	9.97	**<0.001**
Employed part-time	76.44 ± 14.28		
**Number of children**			
0 - 2	85.12 ± 4.76	16.29	**<0.001**
> 2	72.16 ± 14.00		
**Years of working as a nurse in the hospital**			
1-5 years	75.88 ± 10.36	3.50	**<0.001**
> 5 years	82.04 ± 8.68		
**Department working as a nurse in the hospital**			
Emergency department	81.76 ± 5.88	2.03	**<0.001**
Intensive care unit	78.12 ± 12.04		
Other departments	75.32 ± 12.60		
**Working hours per day**			
≤8 hours	79.52 ± 8.18	0.24	0.868
> 8 hours	76.72 ± 14.50		
**Average number of patient are cared for per day**			
≤ 5 patients	76.44 ± 9.66	0.84	0.472
> 5 patients	79.52 ± 8.18		
**Current job rotation status**			
Morning shift	77.84 ± 10.56	3.94	**<0.001**
Afternoon shift	75.72 ± 11.78		
Night shift	82.04 ± 7.62		
**Job position**			
Licensed practical nurse	79.52 ± 8.92	14.26	**<0.001**
Nurse practitioner	73.36 ± 6.94		
**How satisfied are you with your teammates in your working environment?**			
Always	79.80 ± 9.70	6.26	**<0.001**
Never	68.60 ± 13.08		
Sometimes	76.16 ± 11.09		
**Status of being appreciated by superiors**			
Always	80.36 ± 8.57	14.37	**<0.001**

However, several factors demonstrated notable associations with coping mechanisms. Employment status significantly influenced coping strategies, with full-time employed nurses reporting a higher mean score (80.64 ± 8.40) compared to part-time employed nurses (76.44 ± 14.28), with a p-value less than 0.001. The number of children also played a significant role, with nurses having more than two children exhibiting a lower mean coping mechanism score (72.16 ± 14.00) compared to those with 0-2 children (85.12 ± 4.76), with a p-value less than 0.001.

Moreover, years of experience as a nurse, department worked in, current job rotation status, job position, satisfaction with teammates, and feeling appreciated by superiors all showed significant associations with coping mechanism strategies. Nurses with over 5 years of experience had a higher mean coping mechanism score (82.04 ± 8.68) compared to those with 1-5 years of experience (75.88 ± 10.36), with a p-value less than 0.001. Similarly, nurses working in the Emergency department reported a higher mean coping mechanism score (81.76 ± 5.88) compared to those in other departments. Additionally, nurses who reported being always satisfied with their teammates or always appreciated by their superiors exhibited significantly higher coping mechanism scores compared to their counterparts. These findings highlight the multifaceted nature of coping mechanisms among nurses and underscore the importance of considering various socio-demographic and work-related factors in understanding and promoting effective coping strategies within nursing practice.

## Discussion

The COVID-19 pandemic has posed unprecedented challenges to healthcare systems worldwide, placing immense pressure on frontline healthcare workers, particularly nurses, who play a pivotal role in patient care 5, 22, 32. Despite the crucial role nurses play, there remains a dearth of comprehensive research examining the specific socio-demographic and work-related factors that influence nurses' perceptions of stress, emotional regulation, and coping mechanisms in the context of the pandemic, especially within the Nigerian healthcare setting. Understanding these factors is essential for identifying areas of vulnerability and implementing targeted interventions to support nurses' well-being and resilience 17, 18, 25, 26. Therefore, this study fills a critical gap in the literature by providing valuable insights into the factors influencing nurses' experiences during this challenging time, ultimately contributing to the development of evidence-based strategies to support nursing professionals and optimize patient care outcomes.

The study revealed several noteworthy results regarding the socio-demographic and work-related factors influencing nurses' perceptions of stress, emotional regulation, and coping mechanisms. Notably, while gender, age, and religion did not exhibit significant associations with these outcomes, factors such as educational attainment, number of children, years of experience, department worked in, working hours per day, job position, and satisfaction with teammates emerged as significant influencers.

Regarding perceived stress levels among nurses, findings indicated that nurses with an associate or bachelor degree, those with more than two children, those with over 5 years of experience, those working in the Emergency department, those on night shifts, licensed practical nurses, and those reporting dissatisfaction with their work environment experienced higher stress levels. These findings align with existing literature, which highlights the impact of workload, department dynamics, and job satisfaction on nurses' stress levels^6^, ^21^, ^26^, ^33^.

Furthermore, the study uncovered important insights into nurses' emotional regulation strategies. While gender, age, and religion did not significantly influence emotional regulation, factors such as the number of children, years of experience, working hours per day, average number of patients cared for per day, job position, and satisfaction with teammates were associated with differences in emotional regulation scores. These findings underscore the importance of considering individual and situational factors in understanding nurses' emotional experiences and coping strategies^3^, ^21^, ^33^-^35^.

In terms of coping mechanisms, the study identified several significant influencers, including employment status, number of children, years of experience, department worked in, job rotation status, job position, satisfaction with teammates, and feeling appreciated by superiors. These findings emphasize the multifaceted nature of coping mechanisms among nurses and highlight the importance of organizational support and interpersonal relationships in fostering effective coping strategies^9^, ^17^.

The findings of this study align with and expand upon existing literature on the psychological well-being of healthcare workers during the COVID-19 pandemic 6, 23, 32, 36, 37. Similar to research conducted in other contexts, such as Yasin et al. 23, and Elneblawi et al. 6, this study found that high levels of perceived stress and challenges in emotion regulation were prevalent among nurses, exacerbated by long working hours and limited resources. However, this study uniquely highlights the cultural context of Nigerian nurses, whose coping strategies and emotional responses are influenced by specific socio-cultural factors. Cultural values and communal support structures play a significant role in shaping how Nigerian nurses manage stress and regulate emotions, reflecting differences observed in similar studies conducted in other regions^23^. For instance, the emphasis on collective resilience and community-based coping strategies in Nigerian culture may impact how nurses navigate the stressors associated with the pandemic, providing a richer understanding of their emotional experiences compared to studies from Western contexts. This cultural perspective underscores the need for tailored interventions that consider local values and practices to effectively support the mental health of healthcare workers in diverse settings 6, 12, 13, 23.

The results of this study contribute to our understanding of the challenges faced by nurses during the COVID-19 pandemic and underscore the need for tailored interventions and support mechanisms to promote nurses' well-being and resilience 12, 13, 21, 33. By addressing factors such as workload, departmental dynamics, and job satisfaction, healthcare organizations can create supportive environments that enable nurses to cope effectively with stress and emotional difficulties, ultimately enhancing patient care outcomes^36^, ^38^.

In conclusion, this study provides valuable insights into the socio-demographic and work-related factors influencing Nigerian nurses' perceptions of stress, emotional regulation, and coping mechanisms during the COVID-19 pandemic. By integrating these findings with existing literature, healthcare organizations can develop targeted interventions aimed at promoting nurses' well-being and resilience amidst challenging circumstances. Based on the findings of this study, several targeted interventions are recommended to support the mental health and well-being of Nigerian nurses. First, implementing comprehensive mental health support programs within healthcare settings is crucial 39-41. These programs should include regular mental health screenings, access to counseling services, and stress management workshops tailored to the specific challenges faced by nurses. Additionally, enhancing the availability and quality of PPE and improving working conditions, such as reducing excessive working hours and providing adequate breaks, can alleviate some of the stressors identified. Establishing peer support networks and mentorship programs can also foster a sense of community and collective resilience, helping nurses to cope with the emotional demands of their roles. Furthermore, integrating cultural sensitivity into mental health interventions is essential, ensuring that support strategies align with local values and practices. By addressing these areas, healthcare institutions can create a more supportive environment that promotes the psychological well-being of Nigerian nurses and enhances their capacity to manage the demands of their profession effectively.

### Study İmplications and Recommendations

The findings of this study have several implications for nursing practice, policy, and future research. Firstly, the identification of socio-demographic and work-related factors associated with nurses' perceived stress, emotional regulation, and coping mechanisms provides valuable insights for healthcare institutions and policymakers. By understanding these factors, healthcare organizations can develop targeted interventions aimed at mitigating stressors and fostering supportive work environments for nurses. For instance, initiatives focused on reducing workload, providing adequate staffing levels, offering mental health support services, and promoting effective communication channels between management and staff could help alleviate stress and enhance nurses' well-being 13, 14, 18.

Moreover, the identification of specific factors such as the number of children, years of experience, department worked in, and job position as significant predictors of nurses' emotional regulation and coping mechanisms underscores the importance of addressing individual and organizational-level factors in promoting nurses' resilience 11, 21, 33, 37. Interventions tailored to the unique needs of different nurse subgroups, such as those with caregiving responsibilities or working in high-stress environments like the emergency department, can help optimize coping strategies and enhance overall job satisfaction and retention^15^,^26^.

Furthermore, the findings highlight the need for ongoing professional development and support programs for nurses, particularly in the context of crisis situations such as the COVID-19 pandemic. Investing in training programs focused on stress management, emotional resilience, and effective coping strategies can equip nurses with the tools and resources they need to navigate challenging circumstances effectively^4^, ^21^, ^33^, ^42^.

In addition to practice and policy implications, the study findings have implications for future research directions. Longitudinal studies examining the long-term impact of stress, emotional regulation, and coping mechanisms on nurses' well-being and job performance could provide valuable insights into the trajectory of nursing professionals' experiences over time. Additionally, qualitative research exploring nurses' lived experiences and perceptions of stress, emotional regulation, and coping mechanisms could offer deeper insights into the underlying mechanisms and contextual factors influencing nurses' responses to stressors 3, 8, 10, 12.

Overall, the study's findings underscore the importance of addressing socio-demographic and work-related factors in promoting nurses' well-being and resilience, particularly in the context of the COVID-19 pandemic. By implementing targeted interventions and fostering supportive work environments, healthcare organizations can support nursing professionals in managing stress and maintaining optimal job performance, ultimately contributing to improved patient outcomes and quality of care.

### Study Limitation

This study provides valuable insights into the psychological experiences of nurses, but several limitations should be noted. First, the cross-sectional design inherently limits the ability to establish causal relationships between the identified factors—perceived stress, emotional regulation, and coping mechanisms—and their outcomes. While the study offers a snapshot of these variables at a single point in time, it cannot determine how changes over time influence nurses' mental health. Longitudinal studies are needed to assess how these factors evolve and their impact on nurses' well-being over extended periods. Second, reliance on self-reported data introduces the potential for response bias, including social desirability bias. Participants might have altered their responses based on perceived expectations or social norms, leading to inaccuracies in the data. To mitigate this, future research could incorporate objective measures or triangulate self-reports with other data sources to enhance reliability.

Additionally, the study's focus on specific hospitals in Lagos and Abuja may limit the generalizability of the findings to other regions or healthcare settings. Variations in cultural contexts, healthcare systems, and organizational structures could influence how stress and coping mechanisms are experienced and managed by nurses in different settings. Moreover, although the study aimed for a diverse sample, certain subgroups, such as male nurses or those from minority ethnic groups, may have been underrepresented. Addressing this limitation in future research by ensuring a more representative sample and employing mixed-method approaches could provide a more comprehensive understanding of nurses' experiences.

In summary, while this study offers important insights, future research should consider employing longitudinal designs, expanding sample diversity, and using multi-site studies to enhance the generalizability and depth of findings. Incorporating qualitative methods could also provide a more nuanced understanding of nurses' stress, emotional regulation, and coping mechanisms.

## Conclusion

This study sheds light on the intricate relationship between socio-demographic and work-related factors and nurses' perceived stress, emotional regulation, and coping mechanisms. The findings underscore the multifaceted nature of stressors within the nursing profession and the importance of considering various factors in understanding nurses' well-being and coping strategies. While some factors, such as the number of children, years of experience, and job position, demonstrated significant associations with nurses' stress levels and coping mechanisms, others, like gender and age, did not exhibit significant relationships. These results emphasize the need for targeted interventions aimed at addressing specific stressors and enhancing support systems within nursing environments. By fostering supportive work cultures, providing resources for stress management, and promoting effective coping strategies, healthcare organizations can contribute to the overall well-being of nurses and the delivery of high-quality patient care. Additionally, future research should explore longitudinal studies to further understand the dynamic nature of stress and coping mechanisms among nurses and evaluate the effectiveness of interventions aimed at mitigating stress and promoting resilience within the nursing workforce.
